# IMM-101, an immunotherapeutic agent in clinical development as an adjunctive treatment for pancreatic cancer

**DOI:** 10.1186/2051-1426-2-S3-P83

**Published:** 2014-11-06

**Authors:** Hilary Bilyard, Charlotte Mines, Laura Rosa Brunet, Angus Dalgleish, Frances Macintosh

**Affiliations:** 1 Immodulon Therapeutics Ltd, London, United Kingdom; 2 St. George's University of London, London, United Kingdom

## 

IMM-101 is an immunotherapeutic agent in clinical development as an adjunctive treatment in cancer. It comprises heat-killed whole cell *Mycobacterium obuense *and is being developed by Immodulon Therapeutics Ltd, a biopharmaceutical company dedicated to the discovery and development of immuno-oncology therapies. Cancer immunotherapy is based on the evidence that the host immune system, when appropriately modulated, is able to generate protective responses against malignant cells. Such responses, including both innate and adaptive immune components, may affect the establishment, growth and spread of tumours. Hence, therapies which boost general immunosurveillance (e.g. bacterial preparations) through sustained activation of the immune system provide therapeutic potential. IMM-101 has undergone a Phase I safety and tolerability trial in melanoma (NCT01559819), with a number of patients deciding to continue treatment with IMM-101 (NCT01559818). Two Phase II trials are ongoing in advanced, inoperable pancreatic cancer (NCT01303172) and advanced pre-treated colorectal cancer (NCT01539824).

Here we describe "*A Randomised, Open-Label, Proof-of-Concept, Phase II Trial Comparing Gemcitabine with and without IMM-101 in Advanced Pancreatic Cancer"*. This study, investigating the efficacy of IMM-101 as an adjunctive immunotherapy, allows for 12 cycles of treatment over approximately 12 months. The primary efficacy endpoint is overall survival but safety and tolerability, clinical signs and symptoms of disease, progression-free survival and overall response rate are also recorded. Sera, collected monthly, are available for measurements of tumour burden markers, immunological status and relevant mediators, in an effort to identify potential prognostic factors for future assessment.

Recruitment to the trial with 22 sites in 5 countries finished in July 2013 when 142 patients had been screened and 110 randomised, 75 to the investigational group and 35 to the control arm in line with the planned 2:1 active:control ratio. The treatment phase of the study ended in July 2014, with 12 patients from the active group and 1 from the control group having completed 12 cycles of treatment. The remaining patients withdrew from the study before this time point. Patients completing the study were invited to enter a follow-on study, in which they continued to receive IMM-101 and 12, including 1 control patient, were able to do this. Endpoints will be analysed using the safety and intent-to-treat analysis sets. Final results, which will reveal whether patients receiving the IMM-101 immunotherapeutic product in combination with Gemcitabine have significantly improved overall survival compared to those receiving Gemcitabine alone, are expected to be available early in 2015.

